# Prognosis in moderate-severe traumatic brain injury in a Swedish cohort and external validation of the IMPACT models

**DOI:** 10.1007/s00701-021-05040-6

**Published:** 2021-12-22

**Authors:** Elham Rostami, David Gustafsson, Anders Hånell, Timothy Howells, Samuel Lenell, Anders Lewén, Per Enblad

**Affiliations:** 1 Department of Neuroscience, Neurosurgery, Uppsala University, 752 37 Uppsala, Sweden; 2 Department of Surgical Sciences, Radiology, Uppsala University, Uppsala, Sweden

**Keywords:** Traumatic brain injury, Head trauma, Outcome measures, Recovery, Human studies

## Abstract

**Background:**

A major challenge in management of traumatic brain injury (TBI) is to assess the heterogeneity of TBI pathology and outcome prediction. A reliable outcome prediction would have both great value for the healthcare provider, but also for the patients and their relatives. A well-known prediction model is the International Mission for Prognosis and Analysis of Clinical Trials (IMPACT) prognostic calculator. The aim of this study was to externally validate all three modules of the IMPACT calculator on TBI patients admitted to Uppsala University hospital (UUH).

**Method:**

TBI patients admitted to UUH are continuously enrolled into the Uppsala neurointensive care unit (NICU) TBI Uppsala Clinical Research (UCR) quality register. The register contains both clinical and demographic data, radiological evaluations, and outcome assessments based on the extended Glasgow outcome scale extended (GOSE) performed at 6 months to 1 year. In this study, we included 635 patients with severe TBI admitted during 2008–2020. We used IMPACT core parameters: age, motor score, and pupillary reaction.

**Results:**

The patients had a median age of 56 (range 18–93), 142 female and 478 male. Using the IMPACT Core model to predict outcome resulted in an AUC of 0.85 for mortality and 0.79 for unfavorable outcome. The CT module did not increase AUC for mortality and slightly decreased AUC for unfavorable outcome to 0.78. However, the lab module increased AUC for mortality to 0.89 but slightly decreased for unfavorable outcome to 0.76. Comparing the predicted risk to actual outcomes, we found that all three models correctly predicted low risk of mortality in the surviving group of GOSE 2–8. However, it produced a greater variance of predicted risk in the GOSE 1 group, denoting general underprediction of risk. Regarding unfavorable outcome, all models once again underestimated the risk in the GOSE 3–4 groups, but correctly predicts low risk in GOSE 5–8.

**Conclusions:**

The results of our study are in line with previous findings from centers with modern TBI care using the IMPACT model, in that the model provides adequate prediction for mortality and unfavorable outcome. However, it should be noted that the prediction is limited to 6 months outcome and not longer time interval.

## Background

The leading cause of death in the population under 35 years is traumatic brain injury (TBI), and the death rate is 3.5 times higher than cancer and heart diseases combined [7]. Patients with TBI may suffer years of disability, and this causes enormous economic costs for the individuals, families and society. The combined lifetime economic cost of TBI patients in the USA was estimated to be approximately $76.5 billion (in 2010) [6].

Despite the major improvement of TBI outcome in the acute setting and the advancement in the neurointensive care, the therapeutic interventions and prevention of long-term complications remain a huge challenge [10]. A major challenge has been to assess the heterogeneity of TBI pathology and to predict outcome in a precise way. This would not only support clinical decision-making but also play an important role in patient stratification for different clinical trials and provide reliable comparison of outcomes between different groups of TBI patients. Several prognostic models have been developed over the years, but they have been considered to have poor methodological quality, developed from small samples of patients, and not validated on external populations [15]. In order to overcome these shortcomings, Medical Research Council Corticosteroid Randomisation after Significant Head (CRASH) Trial collaborators developed a model on clinical data from the 10 008 patients recruited [3, 17]. The CRASH model showed high discrimination of probability of a poor outcome with area under curve (AUC) of 0.8. Later, the International Mission for Prognosis and Analysis of Clinical Trials (IMPACT) investigators used The IMPACT database including TBI patients, from eight randomized controlled trials and three observational studies, and developed the IMPACT prognostic calculator [3, 22]. It consists of three modules with increasing complexity; the core model consists of age, Glasgow Come Scale (GCS) motor score and pupillary reactivity; second module adds computer tomography (CT) Marshal score, presence of traumatic subarachnoid hemorrhage (tSAH), and epidural mass to the core model plus hypoxia and hypotension; third module adds hemoglobin and glucose concentrations. Several studies have validated IMPACT model in TBI cohorts and report mortality prediction at 6 months outcome with AUC of 0.6–0.89. [1, 2, 5, 8, 9, 11, 13, 14, 19–21, 23, 24] All report an improved prediction with increased complexity.

IMPACT is based upon outcome prediction using GOSE, which is an 8-grade scale. However, the outcome is usually dichotomized into favorable and unfavorable outcome which limits the more precise prediction of functional outcome of a TBI patient with favorable outcome. A more precise prediction of outcome could provide important guidance in the planning of healthcare resources needed for each individual patient facilitating a more individualized therapy, and more importantly better information to the patients and their relatives. Furthermore, it will aid in stratification of patients for different clinical trials.

In this study, we wanted to first validate the predictive power of the IMPACT prognostic calculator in our TBI cohort and further investigate how the predicted outcome was distributed between the actual 8-grade scale GOSE scores.

## Methods

### Patients and study design

The study was approved by the National Ethical Review Authority (Dnr 2010/138, Ö 19-2010, 2015/224). Informed consent was obtained from the patient, or next of kin if the patient was unable give consent. The Declaration of Helsinki and its subsequent revisions were followed.

Patients with moderate to severe TBI admitted to our neurointensive care unit (NICU) at the Department of Neurosurgery at the University Hospital in Uppsala, Sweden, 2008 to 2020, were screened for this study. The available IMPACT variables were retracted from the Uppsala Traumatic Brain Injury register [12]. Complimentary radiology data, as well as laboratory data for hemoglobin and glucose in plasma, and capillary and blood gas tests within 24 h of trauma, respectively, were extracted from the medical records system used at Uppsala University hospital.

**Fig. 1:**
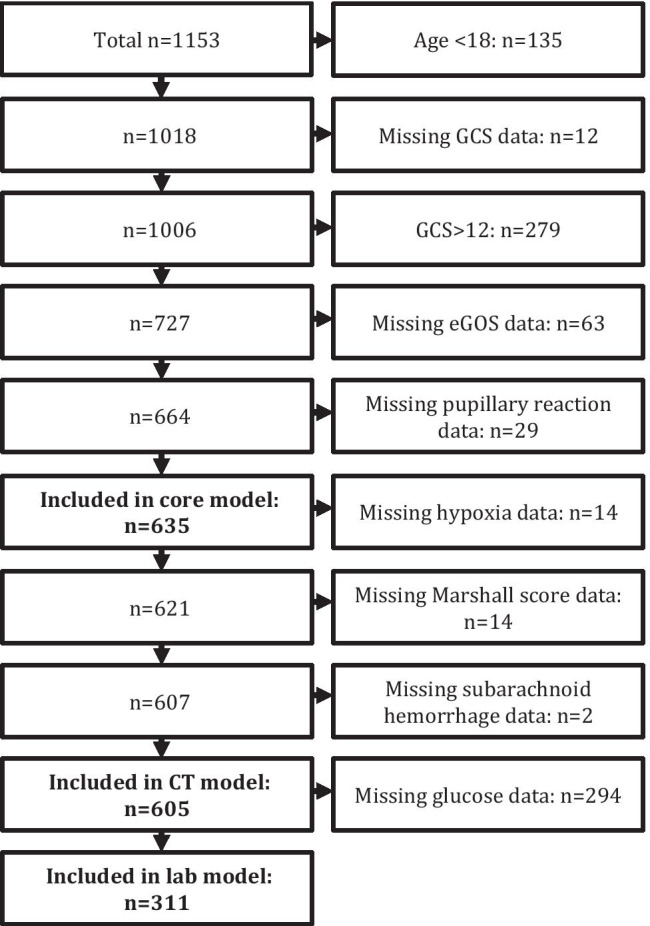
Flowchart describing the number of patients included in each model and criteria leading to exclusion

Patients with an age ≥ 18 and GCS ≤ 12 were included in the study. The flowchart in Figure 1 presents the included patient. We could include 635 patients into the core model of IMPACT. In the CT model, 605 patients could be included (30 patients were excluded due to missing data). Unfortunately, due to a change in medical records system, a significant amount of laboratory glucose data was lost from record, and thus 294 patients were excluded from the laboratory model due to missing data. Due to this, a total number of 311 patients were eligible for inclusion into the laboratory model.

### Outcome

Clinical outcome was assessed at 6–12 months post-injury, by specially trained personnel with structured telephone interviews, using the Extended Glasgow Outcome Scale (GOSE), containing 8 categories of global outcome, from death to upper good recovery.

### Statistics

All data management, statistical calculations, and graphs were made using R (version 3.6.1) [16]. Outcome predictions were made using the IMPACT logistic regression model, and the calculations were verified to correspond to the results generated by the online IMPACT calculator (www.tbi-impact.org), created by the IMPACT investigators. Receiver operating characteristic (ROC) curves and the corresponding area under curve (AUC) were calculated for prediction of mortality (GOSE 1) and unfavorable outcome (GOSE 1–4) using the R package pROC [18]. In order to investigate how the IMPACT prediction (mortality and unfavorable outcome) was distributed between actual GOSE scores, the predicted outcomes were plotted within each GOS- E score. When plotting outcome data, the GOSE values were randomly perturbated ± 0.2 units to avoid overplotting.

## Results

**Fig. 2:**
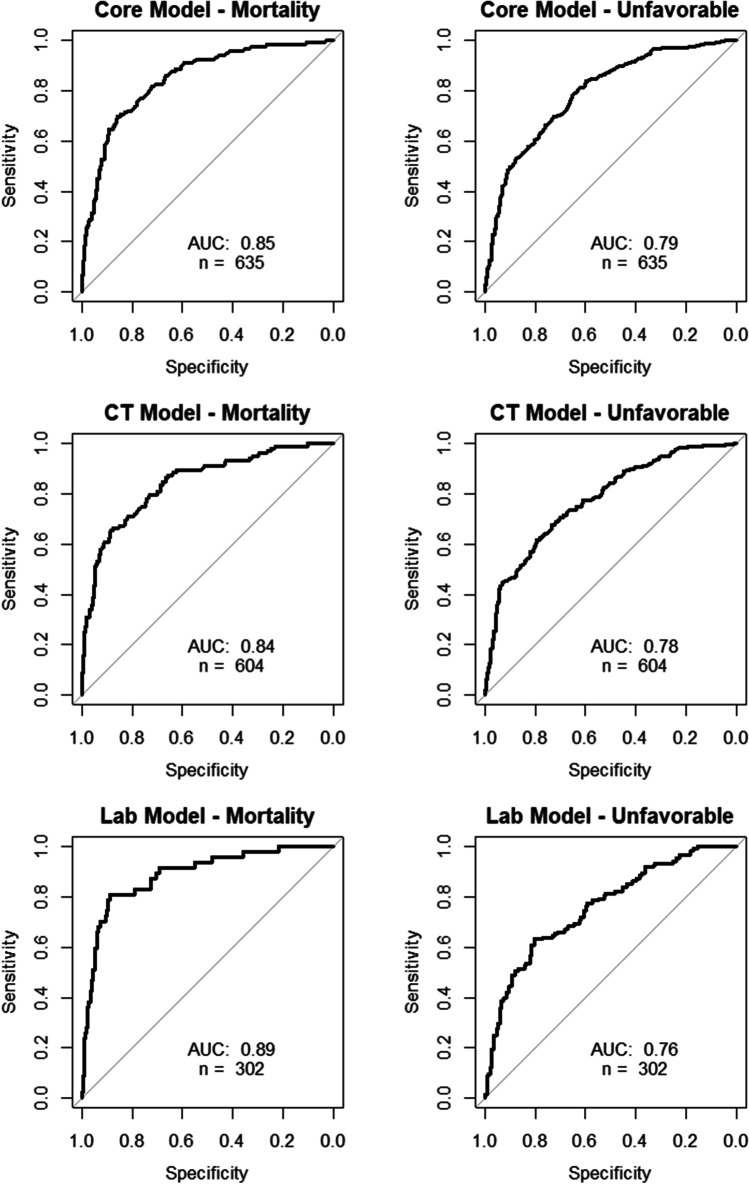
ROC curves for the core, CT, and laboratory IMPACT models

The demographics and characteristics of patients are presented in Table 1. When analyzing the 635 patients with complete parameters for the core model, the core model performed an AUC of 0.85 for mortality. Analysis for unfavorable outcome (GOSE 1–4) showed an AUC of 0.79. Adding CT module to core did not increase AUC for mortality and slightly decreased AUC for unfavorable outcome to 0.78. When the 311 patients having lab parameters (blood hemoglobin and glucose) were included in the model, the AUC for mortality increased to 0.89, while for unfavorable outcome, it decreased to 0.76 (Fig. 2).

**Table 1:** Demographics and site of primary care in the patient cohort. Abbreviations: *ASDH* acute subdural hematoma, *EDH* epidural hematoma, *DAI* diffuse axonal injury, *UUH* Uppsala University Hospital

Parameter	Value	Radiology findings	*n* =	GCS on admission	*n* =	Pupillary reaction	*n* =	Trauma mechanism	*n* =
Number of patients (*n*)	635	ASDH	211	3	41	Both reacting	510	Cyclist hit by vehicle	22
Mean age (years)	53	EDH	37	4	30	One reacting	65	Fall accident	327
Median age (years)	57	Contusion	160	5	28	None reacting	60	Vehicle accident	165
Min age (years)	18	Subarachnoid hemorrhage	44	6	85			Pedestrian hit by vehicle	24
Max age (years)	93	DAI	40	7	55			Assault	19
Male (*n*)	490	Impression fracture	8	7.5	60			Sports accident	16
Male (%)	77	Mixed	116	8	33			Other	62
Female (*n*)	145	Other	13	9	15				
Female (%)	23	Normal	3	9.5	152				
Admitted to UUH (*n*)	127	NA	3	10	9				
Admitted to UUH (%)	20			11	12				
Transferred from local hospital (*n*)	508			11.5	100				
Local hospital (%)	80			12	15

**Fig. 3:**
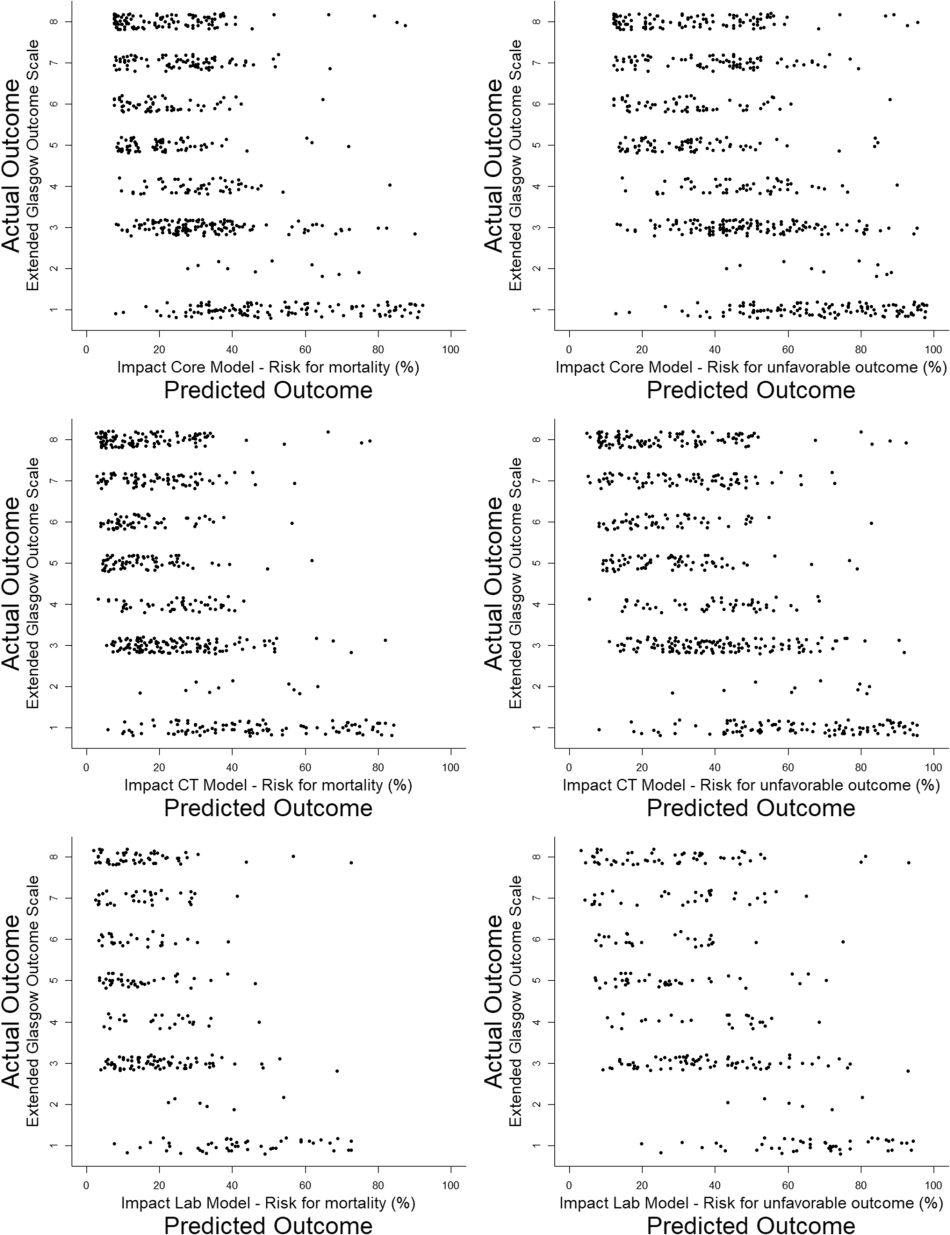
Actual GOSE outcome compared to predicted outcome by the IMPACT models. The dots represent individual patients in their actual GOSE groups compared to percentual risk of mortality or unfavorable outcome respectively. GOSE scale extends from 1 = death up to 8 = upper good recovery. Unfavorable outcome = GOSE 1–4

In order to investigate how well IMPACT could predict the full-scale of GOSE, we compared predicted outcome from the IMPACT models to actual GOSE outcome. As seen in Fig. 3, the core, CT, and laboratory models all generally predicted a low, but still overestimated, risk of mortality in the GOSE 3–8 groups. In the GOSE 1 group, all models predicted a wide range of mortality risk and thus generally overestimated the survival chance of the group. In the prediction of unfavorable outcome, the models produce a spread of outcome prediction in each GOSE group in particular in GOSE 3. Nevertheless, there are clear tendencies that all models correctly predicted low risk of unfavorable outcome in GOSE 5-8 but also incorrectly predicted a low risk in the GOSE 3–4 groups. In the GOSE 1–2 groups, the models correctly predict a much higher risk of unfavorable outcome. It is also notable that a large number of patients ended up in the 40–60% range which does not contribute much to outcome prediction.

**Fig. 4:**
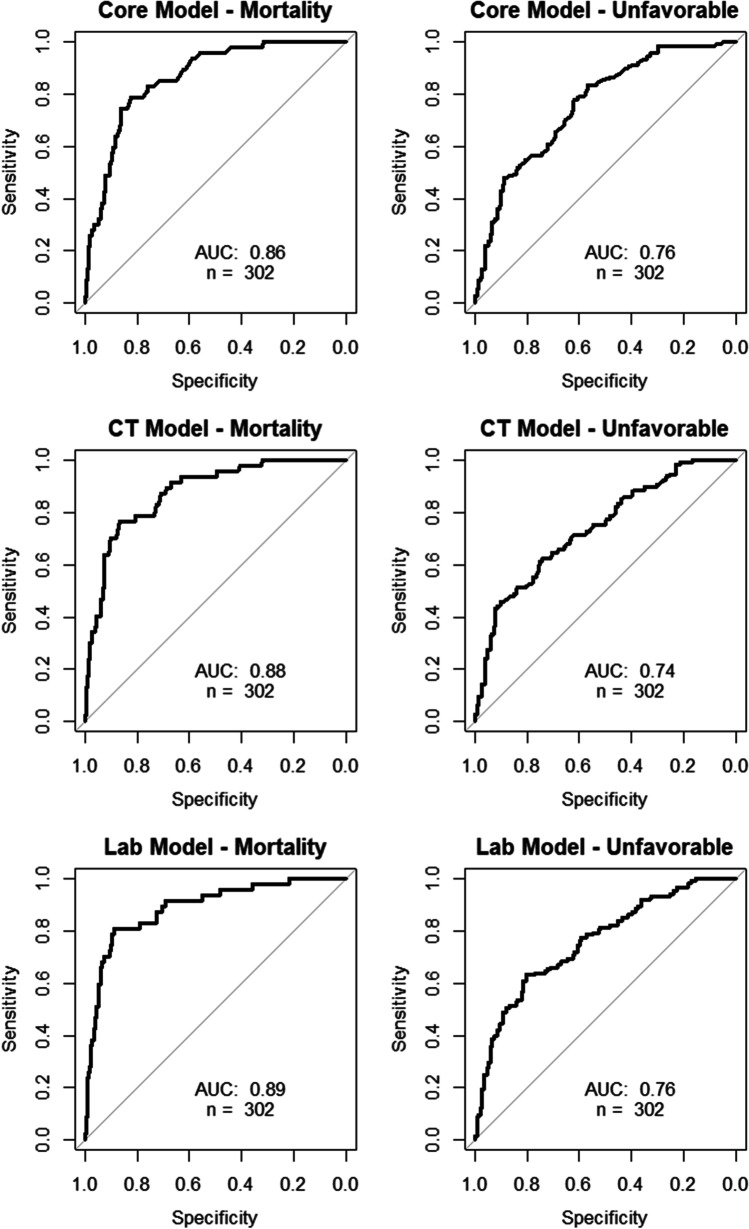
ROC curves for the core, CT and laboratory IMPACT models specifically for the patients included into the CT module

**Fig. 5:**
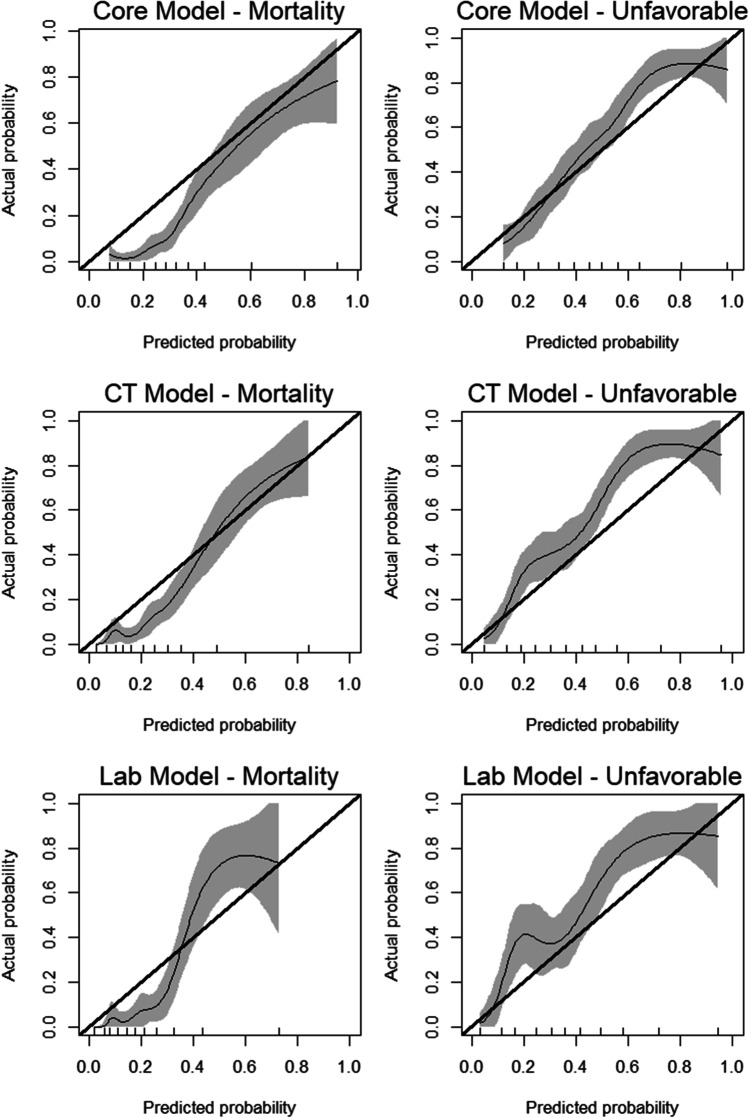
Calibration plots of the core, CT, and lab models. The thin line describes the performance of the models, with the gray area representing two standard errors. The thick line represents the optimal model, where the area over the thick line represents underestimation, and the area under the thick line represents overestimation of risk

Finally, in order to analyze whether the laboratory patient cohort differed from the whole cohort, we also made core and CT module ROC curves specifically for this patient group which yielded similar AUC results (Fig. 4). Finally, calibration plots (Fig. 5) also display fair calibration overall. The core model slightly overestimated the risk of mortality and underestimated unfavorable outcome. The CT model generally performed very well in predicting mortality although there was a slight tendency of underpredicting the risk. Finally, the lab model performed worst, overpredicting the risk of low mortality, underpredicting the risk of high mortality, and generally underpredicting the risk of unfavorable outcome.

## Discussion

Prognostic information of patients suffering from traumatic brain injury is of major interest for several reasons. In the clinical setting, prognostic information in individual cases could improve the planning of individualized healthcare as well as better communication with patient relatives. Furthermore, prognostic information could be utilized in evaluation of clinical studies and the effect of future treatment of TBI. In theory, this could be done prospectively using initial patient data, or retrospectively by placement of patients into high/low-risk categories.

In this article, we validated the IMPACT prognostic model in a Swedish cohort in order to understand the applicability of the IMPACT model in Swedish patients with severe TBI. The IMPACT model is a well-established model for predicting outcome after severe-to-moderate traumatic brain injury, and in our cohort, all three components of the model performed in accordance with previous publications in predicting mortality and unfavorable outcome. Interestingly, there was no apparent difference between the core, CT, and laboratory models, and the best AUC was 0.89. Several studies have previously been performed to externally validate the model. For example, a large external validation on 9036 patients by Roozenbeek et al. (2012) found AUC values of 0.65 to 0.81 and that the model is a credible device in predicting outcome after TBI.[20] Roozenbeek et al. reported in an additional validation of IMPACT on 2513 patients in New York state and found AUC values of 0.79–0.83 concluding that the IMPACT models are generalizable in outcome prediction of TBI. [19] Castaño-Leon AM et al. examined 1301 patients and found AUC values of 0.78 to 0.87 and that the IMPACT models generally underestimated unfavorable outcome, which is also in line with findings in the current study.[1] Similarly, Sun et al. (2016) examined 1124 patients and found AUC values in the range of 0.68–0.71 and concluded that IMPACT underestimated risk in low-risk groups, but contrarily overestimated mortality in the high-risk groups.[23] In a systematic review from 2019, Dijkland et al. concluded that while the model was developed on a large dataset and had a generally adequate discriminative ability, the accuracy of the predictions is variable, and external validation is recommended before clinical implementation. [4] Interestingly, all above studies use GOSE in a dichotomized manner losing the full spectrum of the scale.

In order to assess the full spectrum of GOSE, we investigated the relationship between actual outcome in the full-scale GOSE compared to predicted risk. The predicted mortality was overestimated for GOSE groups 2–8, but in the GOSE 1 group, the model produced a greater range of predicted risk with more chance of survival. There could be several possible reasons for this. Firstly, there are significant differences between the original IMPACT cohort and ours. Sweden as a country is relatively sparsely populated, and Uppsala University Hospital has a geographically large uptake region. The IMPACT model excludes all patients with an initial GCS of 12 and better, and in our cohort, the patients were included depending on GCS upon arrival at UUH NICU as opposed to GCS at arrival to the initial local clinic, which could have been hours to days earlier. As most of the patients in our data came from relocated patients, there is an expected recurring delay in GCS evaluation. However, there is no certain information as to whether the patients generally deteriorated or improved during this delay, and whether this potential change in evaluated GCS significantly affected the predicted risk of the patients. Nevertheless, the laboratory data from these relocated patients was also obtained upon arrival to Uppsala University hospital, and since the AUC value for these patients did not differ in the core and CT module, this could hint that this delay did not cause significant disturbance in IMPACT parameters or prognostic calculation. A second reason for the predicted underestimation of mortality in GOSE 1 group could spring from the fact that the IMPACT models were partially developed using patient data from drug studies in which the exclusion criteria could differ from ours. It is conceivable that patients that were included in this study would have been deemed non-treatable and excluded from the drug studies that IMPACT was based upon. Finally, it is worth mentioning that 6-month mortality depends on several care-giving instances including pre-hospital care, treatment in the intensive care unit, as well as rehabilitation care, all of which could differ from the original IMPACT cohort.

Looking at the predicted unfavorable outcome, it was correctly predicted to be low in GOSE groups 5–8, but the model underestimates risk in the GOSE 3–4 groups and more correctly predicts an unfavorable outcome in GOSE groups 1–2. This means that the included parameters of the model more correctly predict if the outcome is death or a vegetative state but has poorer discriminative ability into placement into the severe and moderate disability groups. However, it should be noted that a large group of patients end up in the range of 40–60% risk of mortality or unfavorable outcome in particular in the core model, which in reality does not contribute to any outcome prediction in real practice since it is almost like “tossing a coin” for these patients.

It would be highly valuable to identify these patients where IMPACT calculator shows poor performance and if they share some common elements such as poor autoregulation and/or genetic vulnerability. Interestingly, all three modules produced similar results in predicting both mortality and unfavorable outcome. In line with previous studies, the CT and laboratory models slightly increased AUC, but this was not the case for unfavorable outcome.

One must be cautious in applying prediction models intended for large cohorts in an individual patient setting. The goal of prediction models might be evaluating the outcome of different cohorts and the care of different centers, but they could also provide insight into whether individualized interventions could improve outcome. A prediction model could also identify patients with unexplainable outlier outcomes that could be of interest for further examination, for example, to find out if there is an unknown reason some patients estimated to have a poor outcome fares much better than predicted.

Furthermore, prognostic information could advance our understanding of the pathophysiology of TBI. By adding novel parameters to the prognostic calculators, such as genetics, and analyzing whether this information impacts the calibration and discrimination of the calculator, it could be possible to analyze the relevance of other pathological and physiological findings and their impact on the prognosis of TBI. This would also mean that the parameters of a prognostic calculator are dynamic and that future novelties and discoveries of the pathophysiology of TBI could impact the calculator and vice versa. In the future, this could lead to more individualized healthcare, where novel parameters could impact the clinical management and treatment of patients.

In conclusion, we report that the IMPACT model produces similar prediction values of mortality at 6 months in our TBI cohort as previously reported. However, the model shows poorer discriminative ability for the rest of GOSE spectrum. Future studies may identify additional important variables such as autoregulation and genetics that can be incorporated into new prognostic models using AI in order to predict the outcome following TBI with more precision targeting the full spectrum of GOSE.
